# Battery-Free and Noninvasive Estimation of Food pH and CO_2_ Concentration for Food Monitoring Based on Pressure Measurement

**DOI:** 10.3390/s20205853

**Published:** 2020-10-16

**Authors:** Thanh-Binh Nguyen, Trung-Hau Nguyen, Wan-Young Chung

**Affiliations:** 1 Department of Electronic Engineering, Pukyong National University, Busan 48513, Korea; binhthanh0608@gmail.com; 2 Faculty of Applied Science, Ho Chi Minh City University of Technology–Vietnam National University, Ho Chi Minh City 72506, Vietnam; haunguyen85@pukyong.ac.kr

**Keywords:** near-field communication, battery-free food monitoring, energy harvesting, smart sensor tag, food pH extraction

## Abstract

In this paper, we developed a battery-free system that can be used to estimate food pH level and carbon dioxide (CO_2_) concentration in a food package from headspace pressure measurement. While being stored, food quality degrades gradually as a function of time and storage conditions. A food monitoring system is, therefore, essential to prevent the detrimental problems of food waste and eating spoilt food. Since conventional works that invasively measure food pH level and CO_2_ concentration in food packages have shown several disadvantages in terms of power consumption, system size, cost, and reliability, our study proposes a system utilizing package headspace pressure to accurately and noninvasively extract food pH level and CO_2_ concentration, which reflection food quality. To read pressure data in the food container, a 2.5 cm × 2.5 cm smart sensor tag was designed and integrated with near-field communication (NFC)-based energy harvesting technology for battery-free operation. To validate the reliability of the proposed extraction method, various experiments were conducted with different foods, such as pork, chicken, and fish, in two storage environments. The experimental results show that the designed system can operate in a fully passive mode to communicate with an NFC-enabled smartphone. High correlation coefficients of the headspace pressure with the food pH level and the headspace CO_2_ concentration were observed in all experiments, demonstrating the ability of the proposed system to estimate food pH level and CO_2_ concentration with high accuracy. A linear regression model was then trained to linearly fit the sensor data. To display the estimated results, we also developed an Android mobile application with an easy-to-use interface.

## 1. Introduction

Near-field communication (NFC) is a technology that allows connecting two devices for data exchange over a short distance. NFC consists of a reader and a tag, operating at the frequency of 13.56 MHz. The tag can be operated in active, semi-passive, or passive modes depending on its power supply. When the tag is placed in a magnetic field generated from the reader antenna, the tag antenna harvests energy for the tag operation to communicate with the reader [1]. Currently, NFC applications are widespread and prevalent, e.g., in the identification, retail and payment, and transport industries, where simple data such as identification numbers are sent from the tag to the reader through NFC data exchange format (NDEF). In recent years, NFC-based energy harvesting has been studied to expand its usability. In other words, instead of reading an identification number, harvested power is used to power an embedded circuit in a sensor node. State-of-the-art studies used NFC-based energy harvesting for reading temperature [2,3], humidity, pH level [4], and soil moisture [5] via smartphones, which are very popular in daily life.

Food quality is one of the greatest concerns in the world as it is closely related to human health [6]. According to the World Health Organization (WHO), foodborne illness causes 600 million people to fall ill every year globally, with 400,000 people dying including 125,000 children under five years of age. In the United States, foodborne illness is also the culprit for approximately 76 million cases of illness, 325,000 hospitalizations, and 5000 deaths each year [7]. Foodborne illness can be identified with various symptoms, such as headache, abdominal cramps, nausea, dizziness, and diarrhea. Among the different characteristics of food, it is worth noting that freshness has a significant impact on food quality. While being stored, food freshness declines as a function of time and storage conditions. The food contamination process causes changes in food features, such as firmness, tenderness, and color, which reduce the taste of food. Therefore, reliable systems for food monitoring are essential in the food production, delivery, and retail industries to limit food waste and foodborne illness.

Spoilage is a complex deterioration of food caused by both chemical and biological activities that make food unacceptable for human consumption [8]. Food spoilage is a function of time, pre-slaughter, post-slaughter, and storage conditions. Oxidation, attacks from enzymes, and microorganisms are the main factors of food spoilage, causing various phenomena in the food container during storage, such as meat discoloration, slime occurrence, pH level change, and gas emission.

Meat pH level and gas emission seem to be the most common indicators used in conventional systems for food monitoring. The pH level of a live animal muscle is approximately 7.1. After slaughtering, the glycolysis process converts glycogen stored in the meat to lactic acid, which reduces meat pH level and produces various kinds of gases such as hydrogen sulfide (H_2_S), ammonia (NH_3_), and mainly CO_2_. For the measurement of food pH level, it is required to touch the food with electrodes, making it an invasive method. This is very challenging for the deployment of the pH meters in large-scale applications because of the difficulties in terms of power consumption, flexibility, and system cost. On the other hand, although measuring CO_2_ concentration in the container headspace is capable of being applied in battery-free food monitoring, there are also challenges facing this method due to the physical sensing property of the gas sensor. Most gas sensors consume too much power when compared with the harvested power from the ambient environment. Furthermore, a long warm-up period is required after supplying power to get accurate measurements from the sensor. There is undoubtedly a risk of mismeasured data related to CO_2_ concentration, which directly affects the accuracy and reliability of the measurement techniques.

Various automatic systems for food tracking have been proposed and developed, acquiring different sensory data to track the changes in food freshness during storage [9,10,11,12,13,14,15]. The conventional indicators used in these works were CO_2_ concentration, food pH level, and meat color, which are intimately related to food freshness. For instance, a hyperspectral imaging system in the spectral range of 400–1000 nm was proposed in [9] for a monitoring system of red meat (beef, lamb, and pork) color. A colorimetric label was also presented in [10,11,13] to track the food contamination process. The label color changed as the food pH decreased during storage. As reported in [16,17,18,19,20], CO_2_ concentration in the food package can also be measured to monitor food freshness when it is released from rotting meat. However, conventional methods are power-consuming, bulky, or battery-operated. This is a huge challenge for large-scale deployment due to the limited flexibility, high cost, and especially the perilous nature of chemical substances in the batteries if ingested.

To solve the problems faced by conventional systems, battery-free food monitoring is a great alternative. Many studies have been carried out in recent years which utilize energy-harvesting technology for self-powered food monitoring applications. The energy sources in these works were mainly from radiofrequency (RF) waves [16,17,18,19,20], where a sensor tag was designed that is able to harvest energy transmitted from a reader for its operation. Antennas were also designed and fabricated to operate at the frequency of 915 MHz [16] and 13.56 MHz [17]. In [17], the author added a relay resonator to the reader and tag antennas to improve wireless power transmission efficiency. On the basis of the fact that food produces substantial levels of CO_2_ during storage, measuring the CO_2_ concentration generated from rotting food in storage containers could be an indicator of food freshness. However, a disadvantage in these systems is that most gas sensors consume too much power (usually tens of milliwatts). Table 1 provides a comparison of the energy harvested from RF waves. We can see that the harvested power is usually too small compared with the power consumption of the CO_2_ sensor. As a result of this problem, the active period of the sensor tag is very short between long charging processes. Running the sensor for a long warm-up time is also recommended to obtain accurate measurements. Hence, applying CO_2_ sensors in battery-free applications faces the risk of incorrectly measured data, thereby directly affecting the system’s reliability.

In this study, we propose a novel system for battery-free food monitoring with high accuracy. The system is based on NFC-based energy harvesting technology and a new method to extract the food pH level and CO_2_ concentration in the container headspace using machine learning. Food pH level and headspace CO_2_ concentration are indicators of food freshness. They are normally measured manually or using invasive, bulky, and power-consuming systems. This work aims to combine NFC-based energy harvesting and headspace pressure measurement for a battery-free and noninvasive estimation of food pH and CO_2_ concentration. To achieve this goal, experiments with perishable food, such as pork, chicken, and fish, were conducted at room temperature and in refrigerated conditions to obtain data related to temperature, headspace pressure, CO_2_ concentration, and food pH level for 2 days. The correlation of the food container headspace pressure with food pH level and CO_2_ concentration was analyzed to validate the proposed method’s feasibility. A linear regression algorithm was implemented to find the best-fit line for the collected data. NFC-based energy harvesting was applied in this work, enabling the system to work without a battery and to communicate with a smartphone to eliminate the need for a dedicated reader as presented in other works.

The contents of this paper are organized as follows: Section 2 describes the methods for food monitoring based on headspace pressure measurement, and the self-powered operation of the sensor tag using NFC-based energy harvesting. The sensor tag operation analysis, data collection process, and the experimental data in two storage environments are discussed in Section 3. Finally, we conclude the paper in Section 4.

## 2. Methods

### 2.1. Proposed Method for Battery-Free Food Monitoring

The glycolysis process leads to CO_2_ production and a drop in food pH, which cause an increase in the container headspace pressure; this pressure can be calculated using the ideal gas equation [28].

(1) PV=nRT,

where *P*, *V*, and *T* are the air pressure, the volume of the container, and the absolute temperature, respectively; *n* is the number of moles of gas, and *R* is the ideal gas constant. Experiments using different sensors were conducted with pork, chicken, and fish stored at room temperature and in refrigerated conditions to obtain data delated to temperature, CO_2_ concentration, pH level, and air pressure for 2 days. The relationships between the variation of pressure data with CO_2_ concentration in the package and the pH value of the meat were also separately evaluated during the stored days at room temperature and in refrigerated conditions. For each experiment, we stored 200 g of food in a 2 L container. Data were read every 5 min and were used to form a dataset for training and testing the linear regression model.

Linear regression is a supervised machine learning approach used to predict a target value on the basis of independent predictors. With a given dataset, the algorithm aims to find the line that best fits the data. In this study, the model of the linear regression algorithm could be described as follows:

(2) $$h\left(x\right)=\;\theta_{0}+\;\theta_{1}x_{1}+\;\theta_{2}x_{2},$$

where *h*(*x*) is the predicted value, *x*_1_ is the food container headspace pressure value, *x*_2_ is the temperature, and *θ*_0_*, θ*_1_*, θ*_2_ are the weighs of the model. The model cost function was computed as the sum of the squared difference between predicted and actual values.

(3) $$J\left(\theta \right)=\;\frac{1}{m}\overset{m}{\underset{i=1}{\sum }}{\left(h\left(x_{i}\right)-\;y_{i}\right)}^{2},$$

where *J*(θ) is the cost function, *m* is the number of data samples, *h*(*x_i_*) and *y_i_* are the predicted and actual values, respectively. The goal of the algorithm is to find the vector θ that minimizes the cost function.

### 2.2. Techniques for Self-Powered Operation

**Figure 1:**
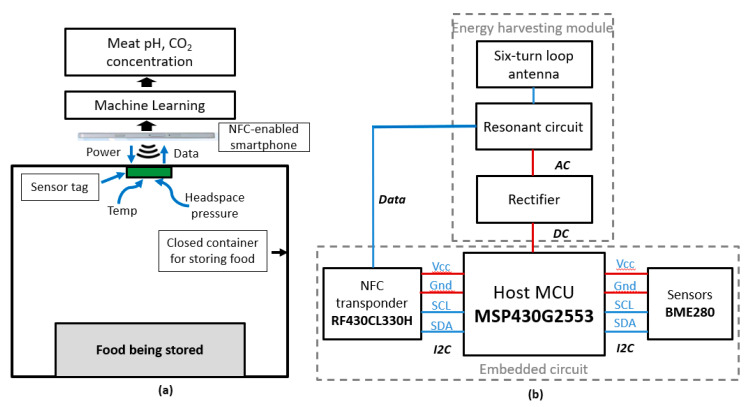
Proposed prototype system: (**a**) block diagram of the proposed system; (**b**) block diagram of the designed sensor tag.

Figure 1 provides a block diagram of the proposed food monitoring system and the design of a smart sensor tag. The system consisted of a closed container to store food, a sensor tag mounted on the inner side of the cap, and an NFC-enabled smartphone operating as a reader. There were two main parts in the sensor tag: energy harvesting and embedded circuit modules for energy harvesting and data collection, respectively. To obtain energy from and transmit data to a smartphone via NFC, we designed a six-turn spiral loop antenna, which was printed on an FR-4 substrate. The antenna dimensions were 2.5 cm × 2.5 cm. Both the coil wire thickness and the spacing between tracks were designed to be 0.25 mm. As the antenna and other components were located on the same printed circuit board (PCB), maintaining a distance of at least 4 mm between the antenna and the ground plane of the PCB was strongly recommended to achieve the best performance from the antenna. We designed the antenna using a simulation tool (Momentum Electromagnetic Simulator, Keysight Technologies, Inc., Santa Rosa, CA, USA) with a simulated inductance of 1.82 µH. Therefore, a tuning capacitance of 75.69 pF was needed to make the antenna resonate at a frequency of 13.56 MHz. The real inductance of the fabricated antenna was then measured using a network analyzer to determine the exact value of the tuning capacitance. Furthermore, we also designed a rectifier with Schottky diodes and a 100 µF capacitor for alternating current (AC) to direct current (DC) conversion.

An important part of the sensor tag is the embedded circuit module, responsible for data collection and transmission. This module had a sensor for exchanging data, a sensor to read various parameters inside the food package, and a microcontroller to control the operation of those sensors. All components needed to be carefully selected on the basis of their technical properties to meet the target of this work.

As mentioned above, NFC is utilized for data communication. We used a dynamic NFC interface transponder (RF430CL330H, Texas Instruments Inc., Dallas, TX, USA) in the current work. This was an NFC Type 4 tag device in which a wireless NFC interface, a wired serial peripheral interface (SPI), and an inter-integrated circuit (I2C) interface were provided for the connectivity between the device and the host micro-controller unit (MCU). The NFC data exchange format (NDEF) message in the static random-access memory (SRAM) could be written and read from an integrated SPI or I2C serial communication interface wirelessly through an integrated ISO14443B-compliant RF interface that supports up to 848 kbps [29].

A low-power microcontroller (MSP430G2553, Texas Instruments Inc, USA) which features a powerful 16-bit reduced instructions set computer central processing unit (RISC CPU), 16-bit registers, and constant generators was used in this study as the MCU to be integrated into the sensor tag for controlling the NFC transponder and the sensor module. The MSP430 family is an ultra-low-power MCU from Texas Instruments that was designed for low-power applications, such as wearable, portable, implantable, and battery-less devices. The operation voltage range of this chip is from 1.8 to 3.6 V and it can be configured at different clock speeds. To achieve the low power consumption of the system, the MCU was configured to operate at 1 MHz, corresponding to a current consumption of 330 µA at 3.3 V in the active mode [30].

To measure temperature, humidity, and headspace pressure inside the food container, an environmental sensor (BME280, Robert Bosch GmbH, Gerlingen, Germany) was selected [31]. This was an integrated sensor developed for applications where size and power consumption need to be minimized. The sensor provides three individual high-linearity, high-accuracy sensors for pressure, humidity, and temperature in an eight-pin metal-lidded 2.5 × 2.5 × 0.93 mm^3^ land grid array (LGA) package, designed for low-current consumption (3.6 μA, at 1 Hz), long-term stability, and high electromagnetic compatibility (EMC) robustness. The sensor features an extremely fast response time for three measurements; in particular, the pressure sensor features exceptionally high accuracy and resolution at very low noise levels. The BME280 sensor supports a full suite of operating modes that provide users with the ability to use the device for power consumption, resolution, and filter performance, which fit the desired application. One of the advantages of this sensor is that all three sensing parts were integrated into a compact surface-mounted package, thus reducing the size of the PCB circuit.

## 3. Results

### 3.1. Sensor Tag Operation Analysis

**Figure 2:**
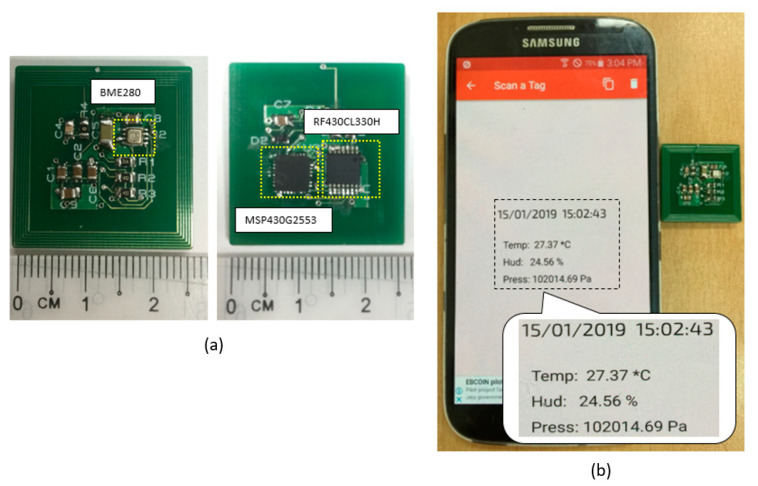
Experiments with the sensor tag: (**a**) top and bottom layers of the printed circuit board (PCB); (**b**) the measured data related to temperature, humidity, and air pressure acquired from the tag using an arbitrary smartphone.

The top and bottom views of the fabricated sensor tag are shown in Figure 2a. With the proposed methods, the sensor tag dimensions could be reduced considerably (2.5 cm × 2.5 cm) compared with sensor tags presented in other work. To demonstrate the operation of the sensor tag in fully passive mode, we used an arbitrary NFC-enabled smartphone to read data from the tag. The result shows that the tag successfully read data related to temperature, humidity, and air pressure from the sensors and sent them to the smartphone via NFC as shown in Figure 2b. The proposed sensor tag was then tested with different smartphones to measure its maximum operating distance. The results show that the sensor tag is able to work with all smartphone models, with the maximum distance ranging from 0 to 9 cm.

### 3.2. Data Collection from Experiments with Food

To obtain data for training and testing the linear regression model, experiments with food at room temperature and in refrigerated conditions were separately conducted. For each experiment, 200 g of food was stored in a 2 L container integrated with a set of sensors for measurements of temperature, headspace pressure, CO_2_ concentration, and food pH level. The container used in this work was a closed rigid box made of glass with a plastic cover. The CO_2_ sensor and pH sensor were mounted beneath the cap of the box. The food container was then sealed with silicon glue to create a closed storage environment as seen in normal food packaging methods. In this experiment, the three sensor modules were powered by an external source through cables for data collection. We also used two Bluetooth low energy (BLE) devices for wireless data transmission. The first BLE module was connected to the sensor modules inside the food package and the other one was connected to a computer. While food was being stored in an air-conditioned room and in the cold room of a refrigerator, the data were read every 5 min. A digital CO_2_ gas sensor (CCS811, Austria Mikro Systeme, Premstätten, Austria), integrated with a metal oxide gas sensor to detect a wide range of volatile organic compounds (VOCs) and an MCU, was used in this experiment. The integrated MCU included an analog-to-digital converter (ADC) and an I2C interface. Food pH level was measured using a spear tip pH sensor (SEN0249, DFROBOT, Shanghai, China) which can be stabbed into a semisolid material such as wet soil and various foods. The pH range of this sensor is from 0 to 10 with an accuracy of ±0.1. In the experiments, the pH meter was kept embedded in the food inside the package.

**Figure 3:**
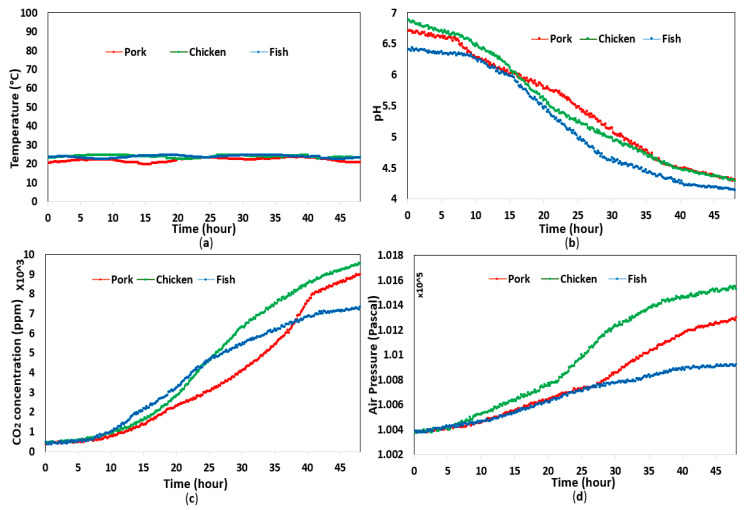
Experimental results with food at room temperature for 2 days: (**a**) temperature; (**b**) pH; (**c**) CO_2_ concentration; (**d**) headspace pressure.

**Figure 4:**
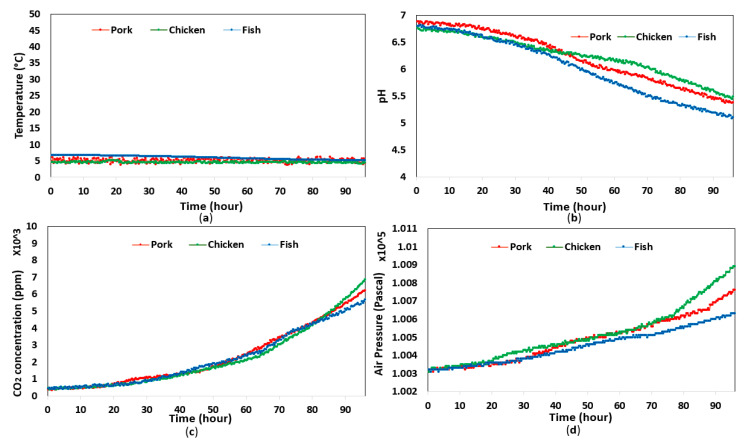
Experimental results with tested food in refrigerated conditions for 4 days: (**a**) temperature; (**b**) pH; (**c**) CO_2_ concentration; (**d**) headspace pressure.

The temperature varied slightly in both experiments; however, they remained at approximately 26 °C and 5 °C for room and refrigerator conditions, respectively. The data related to food pH level, headspace CO_2_ concentration, and pressure demonstrated large variation during the day, whereby pressure and CO_2_ concentration increased gradually, while food pH level decreased due to the degradation in food quality. Figure 3 and Figure 4 depict the data collected from the experiments in both storage conditions, whereby the data related to headspace pressure, CO_2_ concentration, and food pH level displayed different variation rates depending on the type of food and the storage temperature. It is worth noting that the headspace pressure in each experiment increased by thousands of pascals, while the resolution of the sensor used in this study was only 0.18 Pa. Hence, the headspace pressure increase could be accurately measured.

**Figure 5:**
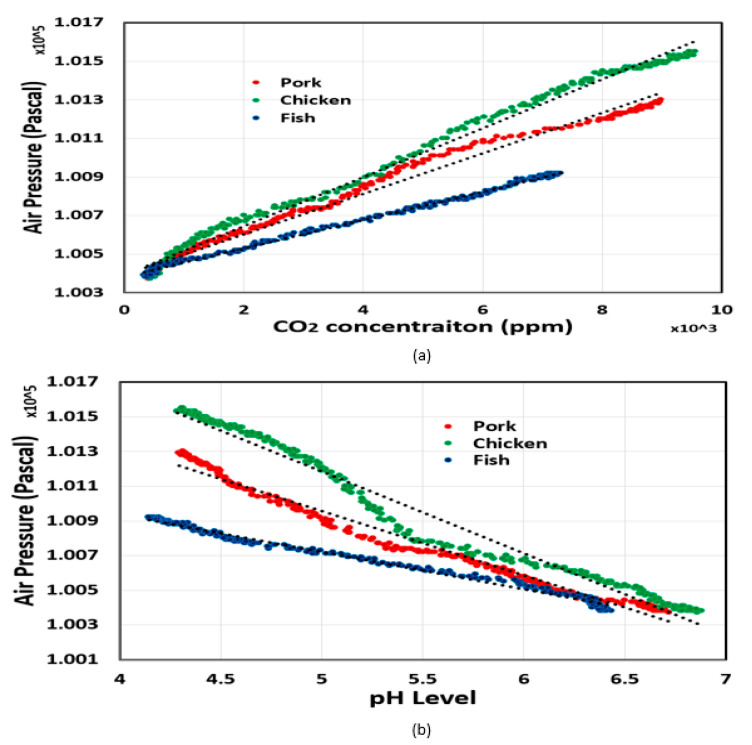
Correlation analysis (**a**) between air pressure and CO_2_ concentration, and (**b**) between air pressure and meat pH level. In this experiment, the tested food was stored at room temperature.

**Figure 6:**
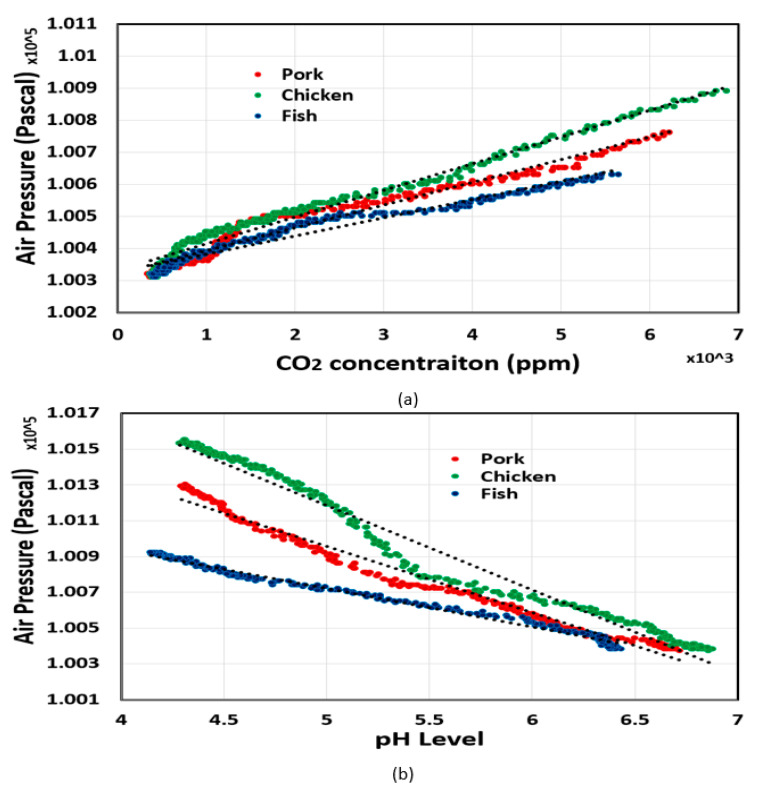
Correlation analysis (**a**) between air pressure and CO_2_ concentration, and (**b**) between air pressure and meat pH level. In this experiment, the food was stored in refrigerated conditions.

The correlation of headspace pressure with CO_2_ concentration and food pH level was analyzed in MATLAB. It was clear that the headspace pressure had a positive correlation with CO_2_ concentration, while the relationship between headspace pressure and food pH level was inversely proportional. As shown in Figure 5 and Figure 6, the correlation coefficients between variables in all experiments were very high, which demonstrates the proposed sensor’s ability to estimate food pH level and headspace CO_2_ concentration from headspace pressure with high accuracy. We also found that the headspace pressure and CO_2_ concentration were linearly proportional. In other words, the CO_2_ concentration could be calculated by measuring the headspace pressure. By contrast, the food pH level had a negative correlation with headspace pressure.

### 3.3. Mobile Application Development

**Figure 7:**
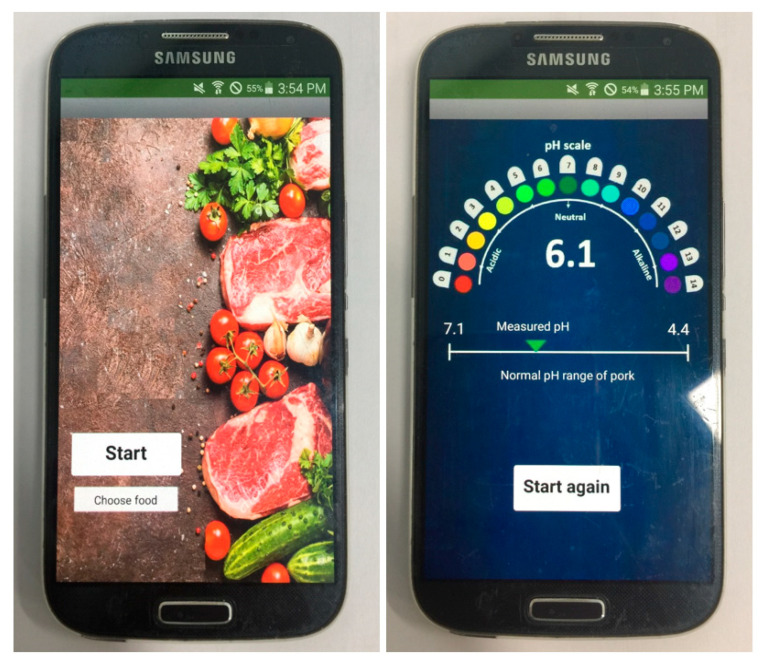
The user interface of the developed Android-based mobile application for food freshness monitoring.

To display the results of the extraction for customers, an Android mobile application was designed that provides an easy-to-use interface. Using the results of the linear regression algorithm mentioned above, the application is able to estimate the chosen food’s pH level and CO_2_ concentration on the basis of the measured temperature and pressure from the sensor tag. Figure 7 shows the interface of the designed application in which users can choose the sort of food to be checked. The results can then be displayed on the screen, including the pH scale for reference, the measured pH of the tested food, the normal pH range of the tested food, and a recommendation message from the system according to this measured pH.

## 4. Conclusions

This paper presented a novel system specifically developed for battery-free food monitoring during storage. A method for extracting food pH level and headspace CO_2_ concentration on the basis of headspace pressure was introduced. The proposed method was evaluated to verify its reliability in food monitoring with different types of food at room temperature and in refrigerated conditions. The feasibility of the low-power headspace pressure extraction method for food freshness monitoring was proven with the high correlation coefficients obtained between the typically invasive food pH level and the CO_2_ concentration in the food package. Validation experiments depicted that the designed smart sensor tag is able to work in fully passive mode to communicate with an NFC-enabled smartphone. Moreover, the power consumption of the sensor tag was reduced to less than 1 mW, compared with tens of milliwatts of power consumption reported in other systems. Additionally, the sensor tag’s compact dimension allows it to be flexibly used in different scenarios. In summary, the proposed system is a promising alternative approach to food freshness monitoring because, with the proposed system, the major drawbacks of other conventional systems, such as high power consumption, poor accuracy, and the need for a dedicated reader, were eliminated.

## Figures and Tables

**Table 1:** Comparison of harvesting systems at different frequency bands. RF, radiofrequency; DC, direct current.

Reference	Frequency (GHz)	Input Power Level of Interest (dBm)	Maximum RF–DC Conversion Efficiency at a Single Frequency (%)	Measured/Simulated Harvested DC Power at the Outdoor Ambient Input Power Level (−15 dBm)
[21]	Dual-band 1.8, 2.2	−30 to −5	55 at −5 dBm	28 µW (measured)
[22]	Four-band 0.9, 1.75, 2.15, 2.45	−15 to 0	60 at 0 dBm	13 µW (measured)
[23]	Dual-band 0.915, 2.45	15 to 0	50 at 0 dBm	17 µW (measured)
[24]	Four-band 0.55, 0.9, 1.85, 2.15	−29 to −10	40 at −12 dBm	38 µW (measured)
[25]	Dual-band 0.915, 2.45	−30 to 0	70 at 0 dBm	26 µW (simulated)
[26]	Single-band 2.45	13 to 20	80 at 13 dBm	Not reported
[27]	Four-band 0.9, 1.8, 2.1, 2.4	−25 to 0	65 at 0 dBm	70 µW (measured)
